# Supplementary figures and images for: Mechanisms of human dynamic object recognition revealed by sequential deep neural networks
Source: PLoS Comput Biol. 2023 Jun 9;19(6):e1011169. doi: 10.1371/journal.pcbi.1011169 (PMC10306191; doi:10.1371/journal.pcbi.1011169)

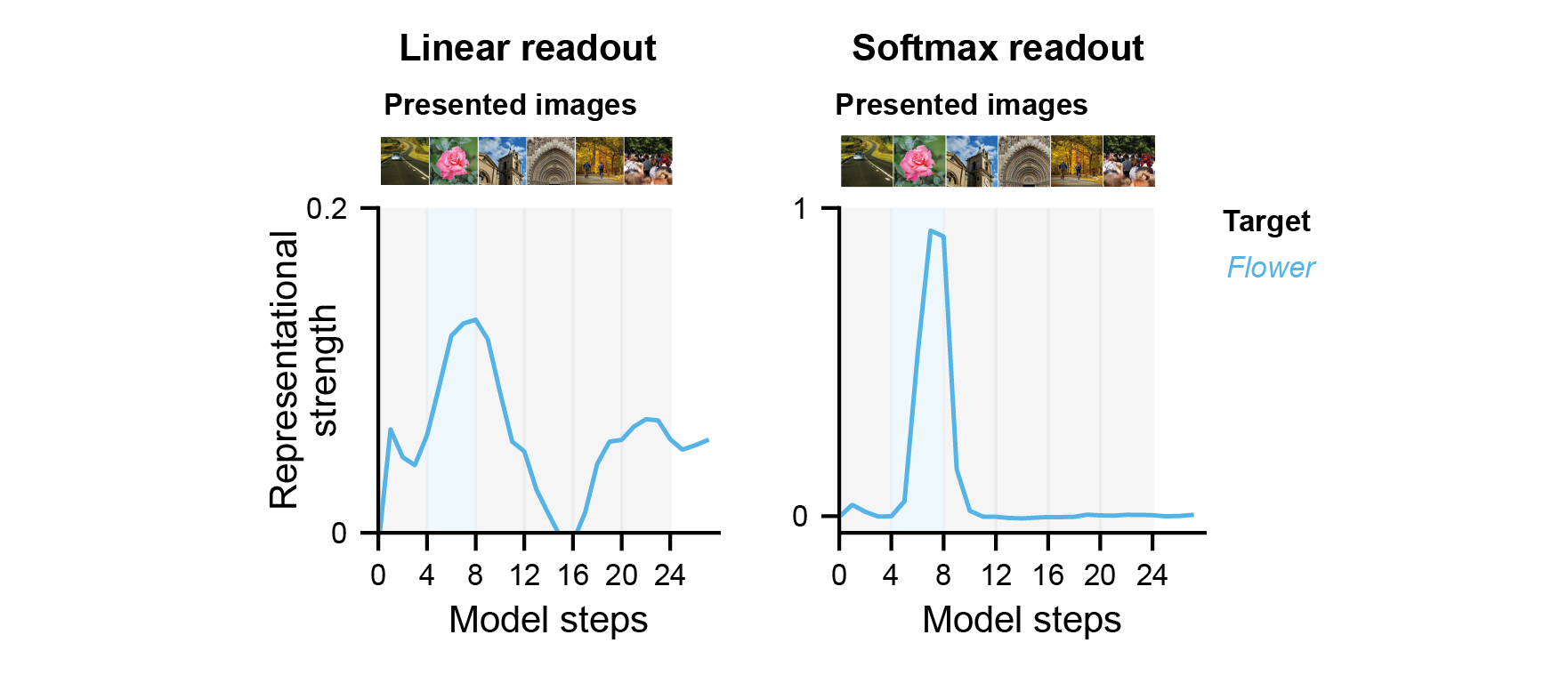

Supplement: S1 Fig — BLnet was optimized with a softmax readout using categorical labels. A softmax operation applies an exponential function to all elements and normalizes them to a sum of 1. This leads to a distribution in which the highest value dominates and as result such a transformation is useful for matching a categorical vector. This principle is illustrated here in the correlation values for a linear and softmax readout. While a linear readout (no skewing toward the maximum value) produced a rather low correlation with the categorical vector (left panel). A softmax readout produces much more extreme correlation values due to its winner-take-all properties, renormalizing across all target classes (right panel). (TIF) [file pcbi.1011169.s001.tif]

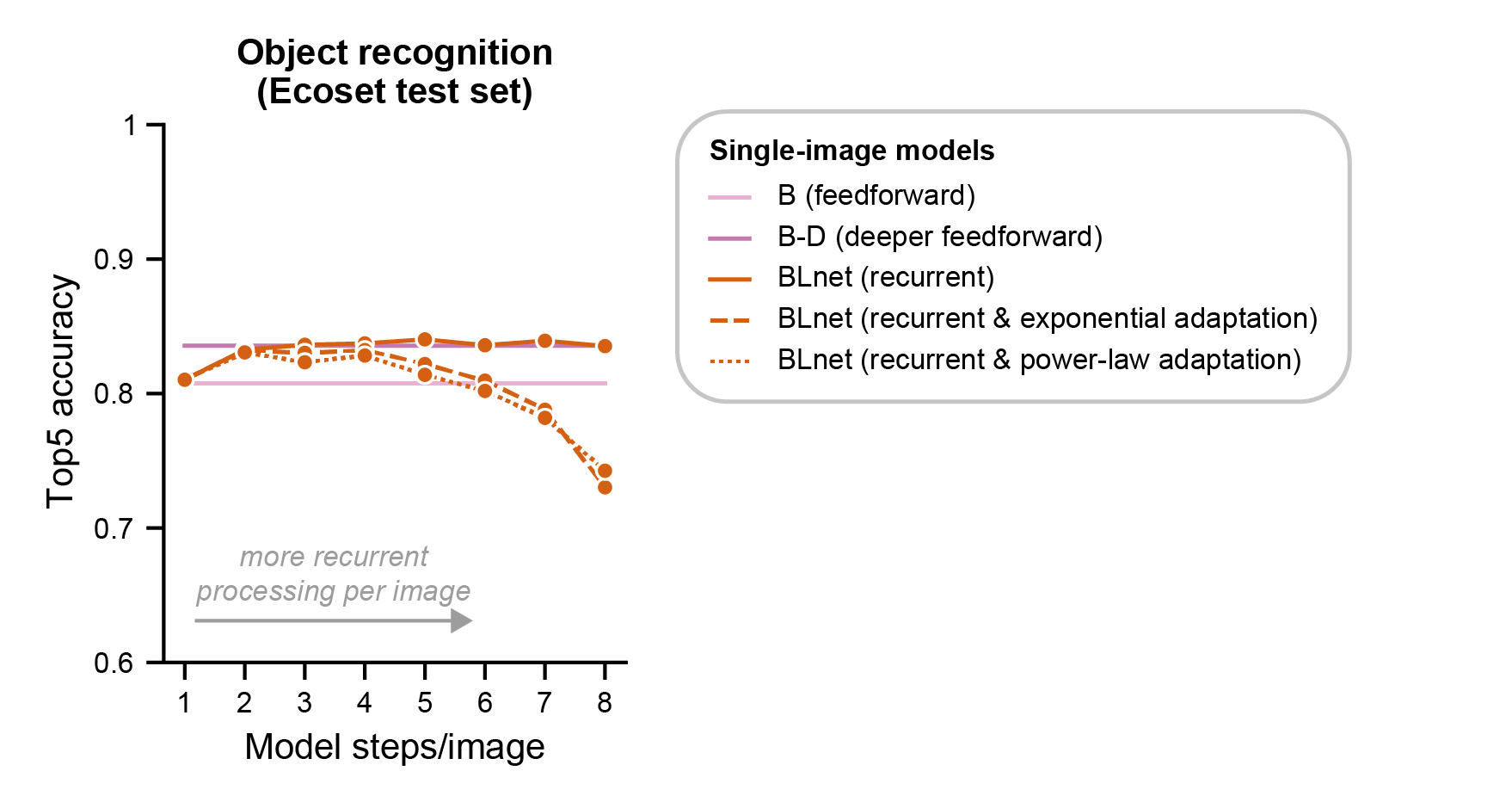

Supplement: S2 Fig — Figure conventions follow those in Fig 3D. In contrast to a sequential recurrent model (BLnext), a single-image recurrent model (BLnet) does not improve its performance after including adaptation mechanisms (i.e., exponential or power-law). (TIF) [file pcbi.1011169.s002.tif]

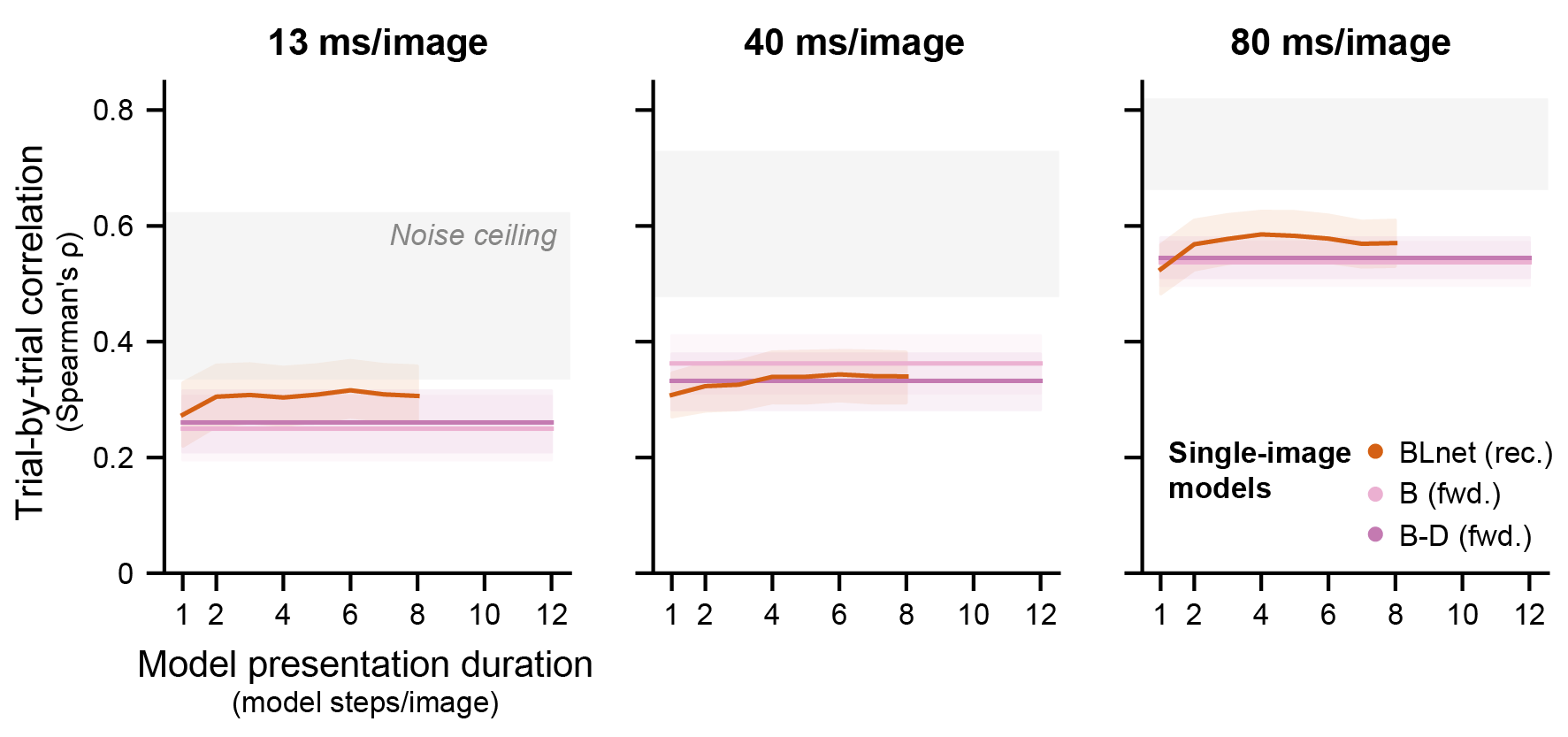

Supplement: S3 Fig — Figure conventions follow those in Fig 5C. (TIF) [file pcbi.1011169.s003.tif]

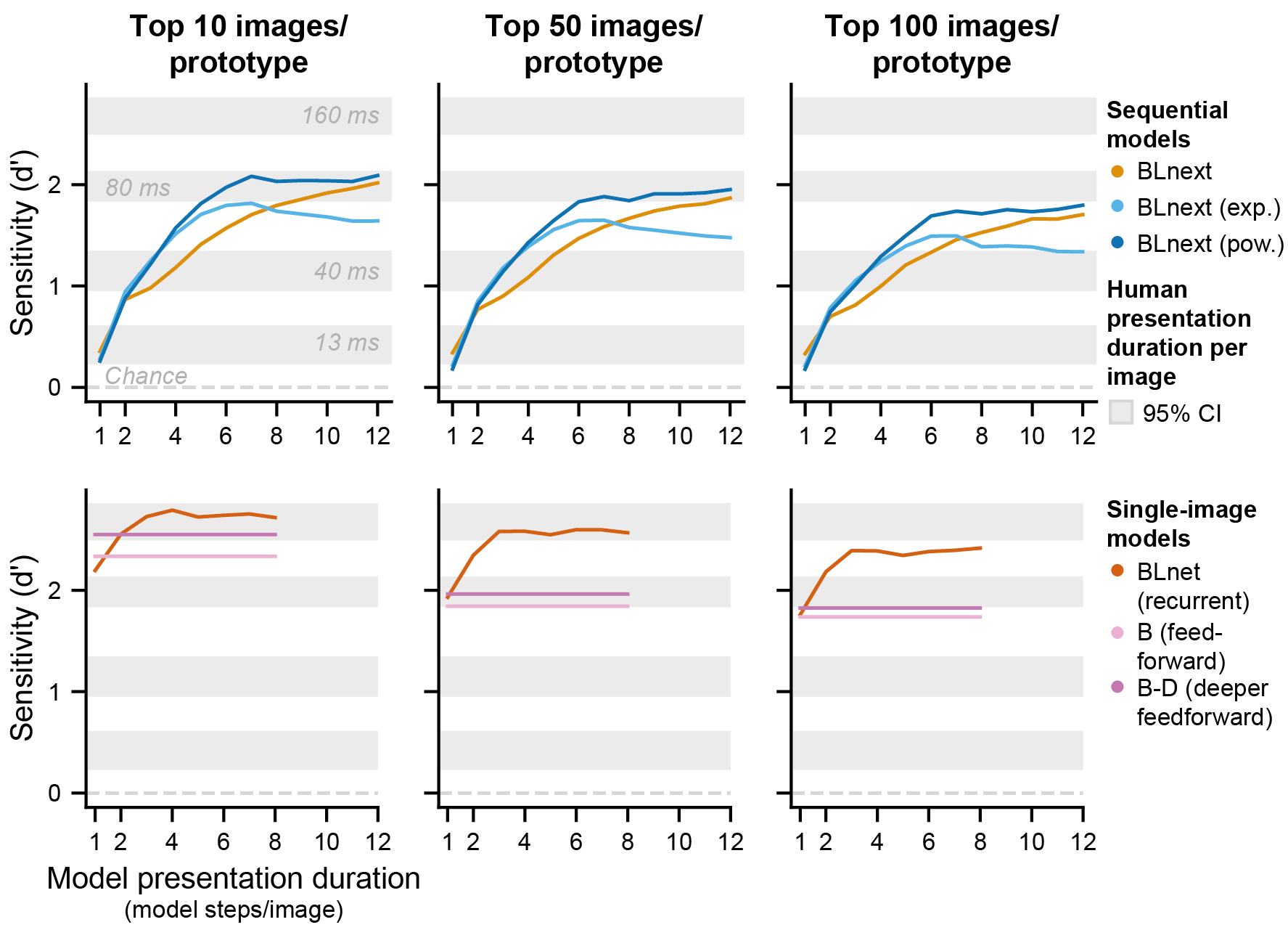

Supplement: S4 Fig — Using prototypes that are based on more exemplar images leads to a reduction in performance levels that affects all model types in a similar fashion. Note that performance is reduced as a result of increasing the number of images because the exemplar images have been ranked with regard to their distance to the mean of the target and foil image. This step was necessary to ensure that the prototype was informative and unambiguous (see Methods). Without such ranking, one would expect performance to increase as a function of exemplars. (TIF) [file pcbi.1011169.s004.tif]
