# Supplementary material for: Mechanisms of human dynamic object recognition revealed by sequential deep neural networks
Source: PLoS Comput Biol. 2023 Jun 9;19(6):e1011169. doi: 10.1371/journal.pcbi.1011169 (PMC10306191; doi:10.1371/journal.pcbi.1011169)
Supplement: Attachment — Submitted filename: ResponseToReviewers.pdf [file pcbi.1011169.s005.pdf]

Amsterdam, April 20, 2022

Dear Dr. Louis, dear Dr. van Vugt,

We hereby submit our revised manuscript "Mechanisms of human dynamic object recognition revealed by sequential deep neural networks" for publication in PLOS Computational Biology. Below is a detailed response to the comments by the reviewers, with the relevant parts of the original comments in italics, and our responses below that.

It was a pleasure to work through the reviewer's valuable suggestions. In particular, based on their recommendation, we added a key control analysis to the manuscript showing that adaptation is particularly beneficial to sequential object recognition. This analysis further strengthens our original conclusions. We have updated our code to also include these new analyses. Furthermore, our revised manuscript now also more clearly describes our approach.

All in all, we believe our paper has improved substantially thanks to the reviews, and we hope it is now suitable for publication in PLOS Computational Biology.

We look forward to your feedback and input on our revised manuscript.

Sincerely,

On behalf of all authors,

Lynn Sörensen

---

***Responses to reviewer 1***

We'd like to thank Dr. Grootswagers for the positive evaluation of our manuscript and his constructive comments. We hope we have fully addressed his comments in the revised manuscript and in our responses below.

***R1.1-2*** *When introducing the BLnet on p5, the authors should justify the rationale for choosing BLnet over any other DNNs.*

*In the next paragraph, BLnext is mentioned but it has not been explained yet what BLnet is and how it is a modification of BLnet*

We thank the reviewer for bringing this to our attention. We have rewritten the following paragraph to address both of these points:

"We here addressed this gap in knowledge by transforming a recurrent DCNN, BLnet [33], optimized for single-image recognition, into a model for sequential object recognition, BLnext. We chose BLnet as our starting point instead of other recurrent DCNNs (e.g., [35]) because BLnet is designed to receive sequences as its input. By applying some modifications to BLnet (e.g., a change to the batch normalization statistics), we obtained a model (BLnext) suitable for sequential object recognition. Using BLnext we could systematically investigate the computational mechanisms critical for object recognition in dynamically changing visual environments."

Introduction – p. 4

***R1.3*** *In Table 1, one may wonder why there is no option for a model that uses dynamic input but without lateral recurrence.*

We agree with the reviewer that this may seem counterintuitive. Indeed, one could cast feedforward models as capable of processing dynamic inputs (i.e., every frame of a dynamic input would simply be treated independently). We have adjusted the terminology in this table to distinguish models that perform recognition on a sequence from those performing recognition on an image-by-image basis (see p. 7).

***R1.4*** *P4 adaption->adaptation*

We have corrected this.

***Responses to reviewer 2***

We would like to thank the Reviewer for their thoughtful and thorough appraisal of our manuscript, which led us to conduct new analyses. These new results have strengthened our paper and its original conclusions. The revised manuscript incorporates all recommendations put forward by the reviewer and we hope to have addressed all concerns in a satisfactory fashion.

***R2.1*** *One major question that I had which was left unaddressed, is how adaptation affects the performance of a recurrent CNN without preceding stimulus. That is, if only one stimulus is presented (e.g. like in the regime where BLnet was trained, or if the target is the first stimulus of a sequence), does adaptation: (a) negatively impact performance, (b) have no real effect, or (c) help?*

We fully agree with the reviewer that based on our previous results we are not able to adjudicate between these scenarios and that this is necessary for supporting some of our conclusions. To further clarify the interplay between lateral recurrence and adaptation, we ran follow-up analyses combining adaptation mechanisms with the single-image *BLnet* model. As per the reviewer's suggestion, these simulations capture how adaptation affects lateral recurrent processing if only a single stimulus is present.

Fig. S2 shows that whereas *BLnet* models with adaptation are indistinguishable from those without adaptation for a few model steps per image, adaptation models show degraded performance when the same image is processed longer (i.e., > 4 model steps). Yet, notably, all models (incl. those with adaptation) still show comparable maximum performance. These results fit with our account that the effects of adaptation become most apparent once the benefits of lateral recurrent processing have been reaped, that is, once a model's recurrent processing becomes repetitive. The result in Fig. S2 also clarifies how including adaptation during sequential processing helps recognition: Once a lateral recurrent model has reached its peak performance, adaptation helps to ready a model for processing new incoming stimuli. Together, these results provide further support for the notion that lateral recurrence and adaptation mutually enhance each other.

We have adapted the manuscript to report this control analysis:

"This finding raises the question whether the performance benefits derived from adaptation mechanisms are specific to sequential object recognition or may also be observed in recurrent models processing one image at a time. Fig. S2 clarifies that the latter is not the case. This suggests that the combination of lateral recurrence and sensory adaptation is particularly beneficial for the challenging task of sequential object recognition."

Results – p. 9

In the process of implementing this follow-up analysis, we encountered a mistake in how the top-5 accuracy was calculated previously for *BLnet* in Figure 3D. We have corrected this mistake and *BLnet* in Fig 3 now shows slightly more modulation over model steps per image.

***R2.2*** *I was curious how a feedforward model with adaptation would perform. Adaptation by itself could increase performance by highlighting target-specific information, but it could also have a detrimental effect.*

We thank the reviewer for raising this interesting question. After careful consideration, we refrained from implementing this additional analysis based on the following reasoning:

In tasks in which sequential stimuli are related (e.g., noise adaptation paradigms), perceptual adaptation to an earlier stimulus can affect the perceptual processing of later stimuli. This process can be mimicked using feedforward models with adaptation mechanisms (Vinken et al., 2020) and for this task, including adaptation leads to an improvement in recognition performance.

Yet, from our understanding, a feedforward model is by definition, the optimal solution for a sequential recognition task in which images within a sequence are unrelated to one another, as is the case in the task that we used in our study. Our task was specifically designed to minimize inter-stimulus interaction effects. Therefore, we do not think that in our case, the performance of a feedforward model with adaptation would be enhanced. All stimulus sequences consisted of unique images and the included images were highly varied and were randomly selected initially. As a result, on average, there was no systematic relationship between any two stimuli and thus only a detrimental effect on a model's performance can be expected in a feedforward model with adaptation.

Although we do not anticipate that a feedforward model may do better with adaptation on our task, if the reviewer or editor feels this analysis would be a critical addition to the paper, we can, of course, run and add it to the paper.

***R2.3*** *Abstract: What result does this sentence refer to (specifically: it sounds like it refers to the same results as the previous sentence): "Importantly, sequential lateral-recurrent integration was also necessary for a model to display a plausible temporal correspondence with human performance".*

We thank the reviewer for pointing this out. The first sentence refers to the model's performance and overall explanatory power. This second sentence refers to the correspondence we observed between human and model performance as a function of presentation durations (Fig. 5D). We have adjusted the wording in the abstract to state this more clearly:

"Importantly, models with sequential lateral-recurrent integration also captured how human performance changes as a function of image presentation durations, with models processing images for a few time steps capturing human object recognition at shorter presentation durations and models processing images for more time steps capturing human object recognition at longer presentation durations."

Abstract – p. 1

***R2.4*** *Fig 2, legend: "representational strength can be computed … as a distance" -> I presume not distance, but similarity/correlation?*

We have corrected this in the manuscript to:

"For pretrained categories, representational strength can be computed for every model and presented image for a given target category presented in the image sequence (flower) as the correlation with the categorical one-hot vector."

Figure 2 – p. 7

***R2.5*** *P10: "We evaluated all sequential models on 500 randomly generated images sequences" -> It would be useful to specify whether the target could be placed at all 6 positions.*

We thank the reviewer for pointing this out. Indeed, the target could be placed in all 6 positions for these experiments. We have updated the manuscript to specify this:

"We evaluated all sequential models on 500 randomly generated image sequences, each consisting of 6 images (all from a different category) and thus 6 targets."

Results – p. 9

***R2.6*** *P10: Fig 5B: Is this for all stimulus durations or for 80 ms only?*

The shown data points stem from human response rates at 80ms/image. We have added a clarification in the legend of the figure:

"The example comparison shows human response rates at 80ms/image and *BLnext (pow.)* model PPCs for 8 steps/image."

Figure 5, p. 13

***R2.7-8*** *P20, Methods, Categorical prototypes: What is a "foil" image?*

*"These images stem from the 2-AFC task..." -> does "these images" refer to the foil images of the previous sentence?*

We agree with the reviewer that this description was incomplete. We have adjusted this paragraph to clarify this:

"To prevent such ambiguity effects from confounding our findings, we used the average representation evoked by a target and a foil image as a marker for semantic relevance. These foil images were chosen to be congruent with the category cue in the original study and were presented as a 2-AFC target identification task directly after the detection task to test whether participants could identify the target images (data not shown or analysed here)."

Methods – Categorical prototypes, p. 18

***R2.9*** *P24, Methods, Single-trial prediction, Predictivity: I was confused by the equation for "RR". I thought RR was calculated for each specific trial, but a target cannot be both absent and present in the same trial. In other words, if a target is present, FP and TN don't apply, and if a target is absent TP and FN don't apply. Or am I missing something?*

We thank the reviewer for catching this. The equation should have specified the number of yes-responses proportional to the number of subjects. We have corrected this in the manuscript to

$$RR = \frac{P}{N}$$

where $P$ refers to the number of yes responses across subjects and $N$ to the number of subjects.

Methods – Predictivity, p. 21

***Responses to reviewer 3***

We want to thank the reviewer for their kind words on our paper and their informative and constructive feedback.

***R3.1*** *About the main finding, that a pretrained recurrent model is affected by changing image presentation rate (just like humans), while a feedforward network isn't, nor is a version of the recurrent model that is artificially reset between image changes: I honestly don't know what else could have been expected here. I agree that (as far as I know) this hasn't been shown before, and that it is therefore worth reporting. But on the other hand, I don't see an alternative outcome that could have made sense.*

We thank the reviewer for sharing their thoughts with us. While embarking on this project, we could foresee some reasons why such models would not result in human-like performance patterns. For instance, we considered whether it may be necessary to optimize models using more dynamic videos to recover this effect. Yet, we found that already models without a specific dynamic training regime produce our pattern of results. We agree with the reviewer that this is a finding worth reporting.

***R3.2*** *2. Related to this, it could have been informative to compare different types of recurrent networks, with different connectivity patterns. For instance, Spoerer et al (2017) had contrasted the BL architecture (lateral connections) with a BT architecture (top-down connections) and a BLT version (both connection types). I think all three types of architectures, if trained on a constant input image, would be affected by changing the input to an RSVP stream. But it's not clear to me which of the three would best match human performance, so this could have been a more interesting question. As far as I know, there is no pretrained version of BT and BLT on the ecoset dataset that could be compared against BLnet, so I understand why authors did not do this, but it could be a worthwhile consideration for future work. It certainly would have made the paper more interesting for me.*

We fully agree with the reviewer that a comparison with a BLT network would be an interesting follow-up avenue. We have added this suggestion to the discussion.

"A key avenue for future studies is to determine how sequential models can be better optimized so that they can account for dynamic object recognition across a wider range of timescales. One potential approach towards achieving this is to train models with other biologically-informed recurrent circuit motifs, such as top-down recurrence."

Discussion, p. 16
